# The Development of Temporal Memory for Complex Events

**DOI:** 10.1111/desc.70160

**Published:** 2026-03-03

**Authors:** Matteo Frisoni, Tiziana Pedale, Michele Capurso, Valerio Santangelo, Carlo Sestieri

**Affiliations:** ^1^ Center for Studies and Research in Cognitive Neuroscience Department of Psychology “Renzo Canestrari” Cesena Campus Alma Mater Studiorum University of Bologna Cesena Italy; ^2^ Department of Philosophy Social Sciences and Education Perugia Italy; ^3^ Department of Neuroscience, Imaging and Clinical Sciences University G. d'Annunzio of Chieti‐Pescara Chieti Italy; ^4^ ITAB Institute for Advanced Biomedical Technologies University G. d'Annunzio of Chieti‐Pescara Chieti Italy

**Keywords:** children's cognitive development, episodic memory, narrative time estimation, temporal cognition, temporal memory development, visuospatial magnitude representation

## Abstract

Remembering when past events occurred is a key component of episodic memory, yet its developmental trajectory remains only partially understood. This study examined how children aged 6 and 10, compared to young adults, recall the timing of events embedded in an 11‐min cartoon. After viewing the cartoon, participants estimated the time of occurrence of short clips extracted from the narrative by placing them on a visual analogue scale representing the episode's duration. They also completed a number line task to assess visuospatial magnitude representation and a chronological reordering task (adapted from the Wechsler Intelligence Scale for Children III), to evaluate narrative chronological organization. Results revealed a clear age‐related improvement in temporal precision. Older children showed almost adult‐like performance and exhibited less bias than younger children. Crucially, differences in visuospatial skills did not fully account for this developmental trend, as controlling for number line and reordering performance did not eliminate age‐related effects. Additionally, only older children showed improved timing estimates for clips rated as more important, indicating the emerging role of narrative structure as a mnemonic scaffold during the school years. Correlational analyses further suggest a developmental shift from spatial to more abstract, narrative‐based representations of time. These findings highlight primary school years as a crucial window for the development of temporal memory in naturalistic contexts and suggest a gradual progression from perceptual to conceptual time representations.

## 1 Introduction

The ability to recall when past events occurred is a fundamental component of episodic memory (Tulving 1983). While previous research has shown that adults are generally able to estimate the time of occurrence of events with considerable accuracy, the developmental course of this ability in childhood remains incompletely understood. Childhood has often been characterized as a “timeless” phase or as a time of “perpetual present” (McEwan 1987), in which temporal markers such as “summer” or “weekend” may be perceived merely as “islands of time” (Friedman 1991). The current study aimed to investigate the development of temporal memory for complex, ecologically valid stimuli across a key developmental window.

### Summary

- Temporal memory improves significantly between ages six and ten, with older children showing performance approaching adult levels in the absence of systematic directional estimation biases.
- 6‐year‐olds exhibit a strong central tendency and overestimate early events, indicating immature mnemonic strategies and heightened temporal uncertainty during early childhood.
- Narrative importance enhances temporal accuracy only in older children and adults, suggesting a developmental shift toward using narrative structure as a memory scaffold.
- A developmental transition emerges from spatial to narrative representations of time, reflecting evolving retrieval strategies and event organization from childhood to adulthood.

### 1.1 A Key Period for the Development of Temporal Memory

Although infants and toddlers demonstrate the ability to recall the order of actions in short event sequences (e.g., Bauer et al. 1998, Bauer and Thal, 1990; Bauer et al. 2000; Lukowski and Bauer 2013), these early competencies are typically regarded as rudimentary and are thought to rely on mechanisms distinct from those supporting mature temporal memory (Bauer and Leventon 2013). By the preschool years, children begin to exhibit an emerging understanding of the past as a discrete temporal category and can make basic distinctions between temporal intervals (Friedman 1992). Nevertheless, children under the age of six often struggle to situate events within broader temporal structures such as days of the week or seasons (Friedman 1991; Friedman et al. 1995). For instance, although 4‐year‐olds may distinguish more from less recent events, they typically fail to anchor these events using conventional time markers (Friedman 1991). Research indicates that temporal knowledge continues to develop throughout the school years, with improvements observed in children's ability to situate autobiographical events in time (Bauer et al. 2007) and to sequence familiar events accurately (Friedman 1986). It is during this developmental window that children may begin to acquire generalized temporal schemas that allow for the reconstruction of when events occurred—processes analogous to those seen in adults (Friedman 1993; Frisoni et al. 2021, 2025).

Despite these advances, empirical research on the emergence and refinement of temporal memory from early to middle childhood remains limited (Pathman, Doydum et al. 2013; Hayne and Imuta 2011). This period, spanning approximately ages 6 to 10, is particularly significant. During these years, children transition into formal education, adopt more structured daily routines, and are increasingly exposed to temporal language and discourse surrounding planning and future‐oriented thinking (Zhang and Hudson 2018). Concurrently, theoretical models suggest that more structured temporal representations, such as a mental timeline, begin to form during this developmental stage. These representations are thought to be shaped by culturally grounded factors, including reading direction, narrative processing, and educational exposure (Boroditsky et al. 2011; Tillman et al. 2018; Autry et al. 2020). As such, the transition from early to middle childhood may represent a crucial phase for the development of reconstructive temporal memory. During this time, children not only engage with formal instruction and routines, and increasingly participate in time‐related discourse and literacy practices, which are believed to scaffold the construction of a mental timeline (e.g., Pathman et al. 2018; Autry et al. 2020). Theoretical models identify this stage as a pivotal point in the development of temporal cognition (Hoerl and McCormack 2019). However, empirical findings remain fragmented. Further research is needed to determine how and when children develop the ability to temporally situate events, particularly in contexts that reflect the complexity and narrative richness of real‐life experiences.

### 1.2 Using Narrative Media to Investigate the Development of Temporal Memory for Complex Events

Narratives, and especially movies, have become a widely adopted tool for studying temporal memory in adults, offering ecologically valid yet experimentally controllable stimuli that reflect how individuals encode and retrieve complex event sequences (Frisoni et al. 2021, 2024, 2025; Chang et al. 2022; Lee et al. 2020; Nastase et al. 2019; Furman et al. 2007). This methodological approach is based on the idea that the cognitive mechanisms engaged during the processing of fictional and real‐life events largely overlap across individuals (Radvansky and Zacks 2014). In contrast, relatively little is known about how young children process age‐appropriate narratives with embedded temporal structures, such as those presented in animated cartoons. There is evidence that, by 18 months of age, infants are sensitive to narrative coherence (Pempek et al. 2010; Richards and Cronise 2000) and can distinguish structured content from scrambled or degraded stimuli (Sonne et al. 2018), suggesting an early receptiveness to narrative form. However, narrative comprehension at this age appears limited. For example, while 2‐year‐olds attend to narrative elements in picture books, their understanding of story structure remains rudimentary (Kaefer et al. 2015). Although children as young as three can recognize causal relationships between static images (Gelman, et al.^1980^), they often struggle to integrate events into coherent temporal sequences (O'Connell and Gerard 1985; Berman and Slobin 1994). Around the age of four, children begin to shift from labeling isolated images to producing more sequential narratives (Berman 1988; Trabasso et al. 1992). Nevertheless, their narratives frequently lack essential components of story grammar, such as goal‐directed actions or clear resolutions (Peterson and McCabe 1983; Beck and Clarke‐Stewart 1998), and show limited microstructural cohesion (Mäkinen et al. 2014).

Between the ages of four and six, children begin to produce narratives that incorporate the core elements of story grammar (Bartlett 1932; Mandler and Johnson 1977). These include initiating events, goal‐directed actions, and resulting outcomes (Trabasso et al. 1981) and are increasingly organized in a coherent chronological order (Peterson and McCabe 1983). Children also demonstrate a growing ability to link sentences cohesively (Peterson and McCabe 1991), to infer missing information, and to identify causal relations within stories (Brown and Murphy 1975; Schmidt and Paris 1978; Zampini et al. 2017). These developments are supported by advances in linguistic competence, including the use of temporal and causal connectives (Lahey 1988), as well as improvements in attentional control, which reduces susceptibility to environmental distractions (Cavallina et al. 2018; Pedale et al. 2021; Pedale et al. 2023). Notably, children tend to prefer narrative material and show better memory for structured stories. However, narrative comprehension remains incomplete during early childhood (Milch‐Reich et al. 1999a; Paris and Paris 2003; Schneider et al. 2006) and only reaches full sophistication in late adolescence or early adulthood (Lever and Senechal 2011; Mäkinen et al. 2013; McCabe and Peterson 1991). Thus, while children may exhibit improved memory for narrative content, their capacity to comprehend overarching storylines continues to develop progressively throughout early and middle childhood.

Moreover, children's recall of narrative content appears closely linked to the causal structure of the story. In a study comparing 4‐ and 6‐year‐olds with adults, participants listened to stories varying in the number of causal connections between events. Although older participants recalled more information overall, events with stronger causal links were remembered more accurately, an effect that strengthened with age (van den Broek, Lorch, and Thurlow 1996). Similarly, Poulsen et al. (1979) found that when children viewed picture stories presented in coherent or scrambled order, 4‐year‐olds focused on isolated scenes while 6‐year‐olds were able to infer causal connections and reconstruct a coherent narrative, resulting in better memory integration. These findings suggest that causal coherence facilitates the construction of structured event representations, which in turn enhances episodic memory performance. Indeed, causal connectivity is considered a core organizing principle of long‐term memory (Radvansky et al. 2005; Radvansky and Zacks 2017) and plays a key role in temporal judgment, particularly in determining the relative order of events (Friedman 1993). Based on this body of evidence, we hypothesize that, in our experimental task (detailed in the paragraph below), events judged as highly important to the narrative, presumably those with greater causal significance, will be recalled with greater temporal precision moving from early to late childhood.

Previous work in adults has combined naturalistic narrative materials with explicit measures of temporal order memory, providing important insights into the cognitive and neural mechanisms supporting temporal organization in episodic memory. In a series of behavioral and neuroimaging studies, Kwok and colleagues showed that temporal order judgments for events embedded in continuous narratives are systematically modulated by temporal distance, event boundaries, and attentional factors at encoding and retrieval (Kwok et al. 2012, 2014; Kwok and Macaluso 2015a, 2015b, 2015c). At the neural level, these studies have consistently identified a distributed network, including medial parietal regions (precuneus and angular gyrus), lateral parietal cortex, and medial temporal structures, as being involved in the retrieval of temporal information from complex events. More recent work has further linked temporal precision and the representation of temporal structure to interactions between the entorhinal–hippocampal system and parietal regions, as shown using both fMRI and electrophysiological approaches (Montchal et al. 2019; Frisoni et al. 2025), as well as to distinct neural signatures associated with subjective aspects of remembering, such as vividness, in the angular gyrus (Zou and Kwok 2022). Together, this evidence suggests that memory for the temporal order of narrative events in adults relies on reconstructive processes supported by a parietal–medial temporal network, which provides a neural context for the present behavioral investigation.

### 1.3 The Present Study

The current study examined the development of temporal memory across two distinct childhood age groups—6‐ and 10‐year‐olds—and evaluated whether primary school children can temporally locate events within a narrative, and how their performance compares to that of young adults. To address these questions, we tested three groups: Italian children aged 6 years (first grade), 10 years (fifth grade), and a control group of young adults. All participants viewed a complete episode (approximately 11 min in duration) of the animated television series Robin Hood. Following encoding, they were asked to indicate the temporal position of several short clips extracted from the episode using a visual analogue scale representing the full timeline of the episode. For each clip, participants also rated its perceived importance within the overall narrative. As a control for alternative explanations of age‐related effects, participants completed two additional tasks: a number line estimation task, which assessed their ability to translate magnitudes into visuospatial representations, and a picture reordering task adapted to evaluate narrative comprehension. The latter served to exclude difficulties in story understanding as a source of errors in temporal placement. All tasks were administered individually using touchscreen devices. We hypothesized a linear developmental progression in temporal memory, with accuracy increasing across age groups, and a marked improvement between ages 6 and 10. Additionally, we expected that older children would rely more heavily on narrative structure to guide temporal judgments, such that events judged as more important to the storyline would also be placed with greater temporal precision.

## 2 Methods

### 2.1 Participants

A total of 115 participants were recruited from three primary schools (Scuola Primaria di Cerbara, Scuola Primaria di Lerchi, and Scuola Primaria di Riosecco), all part of the Direzione Didattica 2°Circolo “Pieve delle Rose” in Città di Castello, Perugia, Italy. Adult participants were recruited from the University of Perugia. All participants reported no prior familiarity with the audiovisual material used in the study. Eight individuals were excluded from the final sample due to average performance (absolute error) more than two standard deviations below the mean on either the Timeline or Number Line task (two adults, three 6‐year‐olds, and three 10‐year‐olds). The final sample thus comprised 107 participants. This included: 36 6‐year‐old children (first grade; 21 female; M age = 6 years 7 months; range: 6 years 1 month to 7 years 3 months), 45 10‐year‐old children (fifth grade; 29 female; M age = 10 years 7 months; range: 10 years 1 month to 11 years 4 months), and 26 young adults (university students; 21 female; M age = 22 years 5 months; range: 20 years 3 months to 26 years 11 months).

With respect to community of descent, the vast majority of participants were of Italian/European descent (>95%), consistent with demographic data from the Umbria region (ISTAT 2023). Regarding socioeconomic status, no individual‐level data were collected; however, based on regional statistics, participants’ families were broadly representative of the local population, with average parental education corresponding to completion of secondary education and average household income within the national middle‐income range (ISTAT 2023).

The appropriate sample size for this study was estimated with G*Power 3.1.9.2 (ANOVA, repeated measures, within‐between interaction), taking into account: a medium effect size of 0.25 [based on previous findings demonstrating the sensitivity of temporal precision indices to experimental manipulations (Frisoni et al. 2021, 2024, 2025)], a power of 95%, a significance level of 0.05, 3 groups, 3 measurements (movie parts), correlation among repeated measures of 0.5, and non‐sphericity correction of 1. This indicated a minimum sample size of 18 participants per group.

Exclusion criteria for children included any diagnosed neurodevelopmental disorders, as reported by parents or teachers. All participants had normal or corrected‐to‐normal vision and were naïve to the purpose of the study. Adult participants provided written informed consent. For children, written parental consent was obtained, and verbal assent was secured from each child. The study was conducted in accordance with the Declaration of Helsinki. Testing was conducted individually and consisted of an encoding phase (∼11 min), a retrieval session, and two control tasks, with a total duration of approximately 40 min. We ensured that none of the participants included in the sample had previously watched the cartoon. Children were informed that participation was voluntary and anonymous, that they could withdraw at any time, and that no school grades would be assigned. Alternative activities (e.g., drawing, homework) were offered for those who chose not to participate. All children who took part provided verbal assent.

### 2.2 Stimuli and Procedure

**FIGURE 1:**
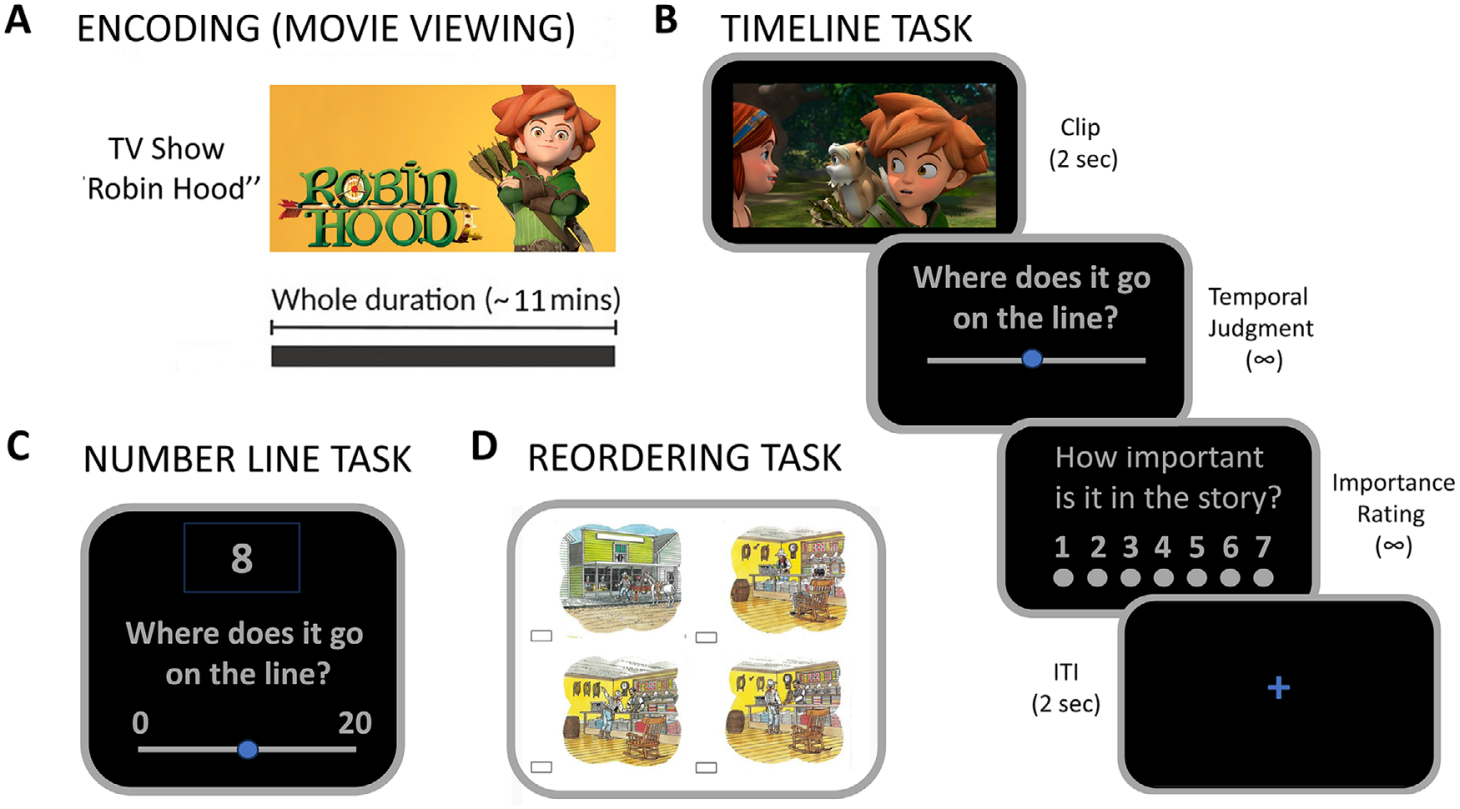
Task structure. (A) In the encoding phase of the Timeline Task, participants watched an episode of the TV series *Robin Hood*. (B) In the retrieval phase, they were asked to estimate the exact position of the video clip extracted from the episode on a VAS representing the episode's full duration. Next, they rated the importance of the clip for the whole narrative on a 7‐point Likert scale. (C) In the Number Line Task, participants were asked to estimate the exact position of each number displayed on a VAS representing numbers from 0 to 20. (D) In the Reordering Task, participants reconstructed the correct sequence of four simultaneously presented images by entering a number beneath each image.

**FIGURE 2:**
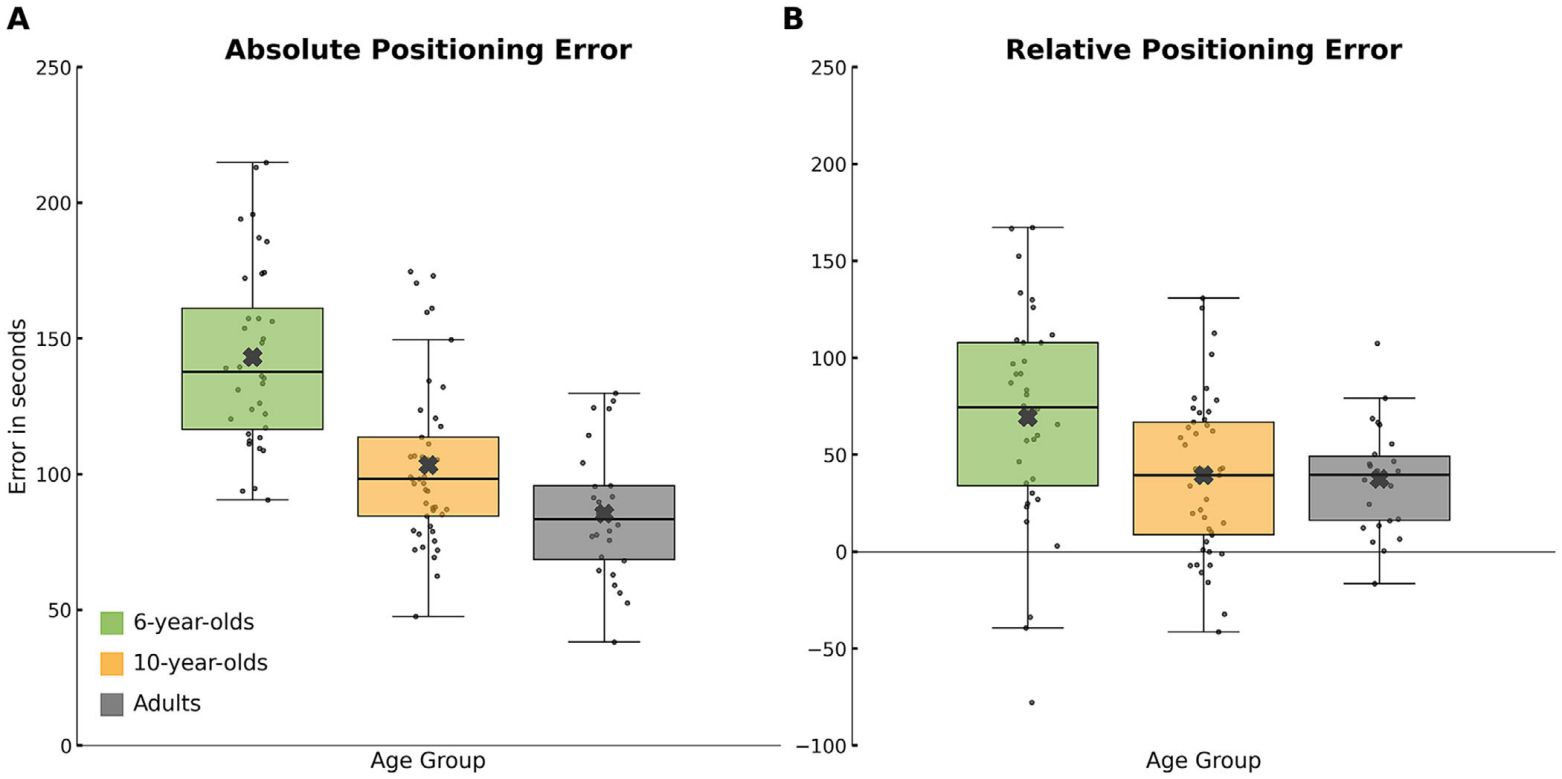
Absolute and relative positioning error in the timeline task by age group. Boxplots display group averages (black crosses) for 6‐year‐olds (green), 10‐year‐olds (orange), and adults (gray), shown separately for absolute error (A) and relative error (B). Each box represents the interquartile range (IQR), with the horizontal line indicating the median; whiskers extend to 1.5 × IQR. Individual data points are shown as jittered black dots.

**FIGURE 3:**
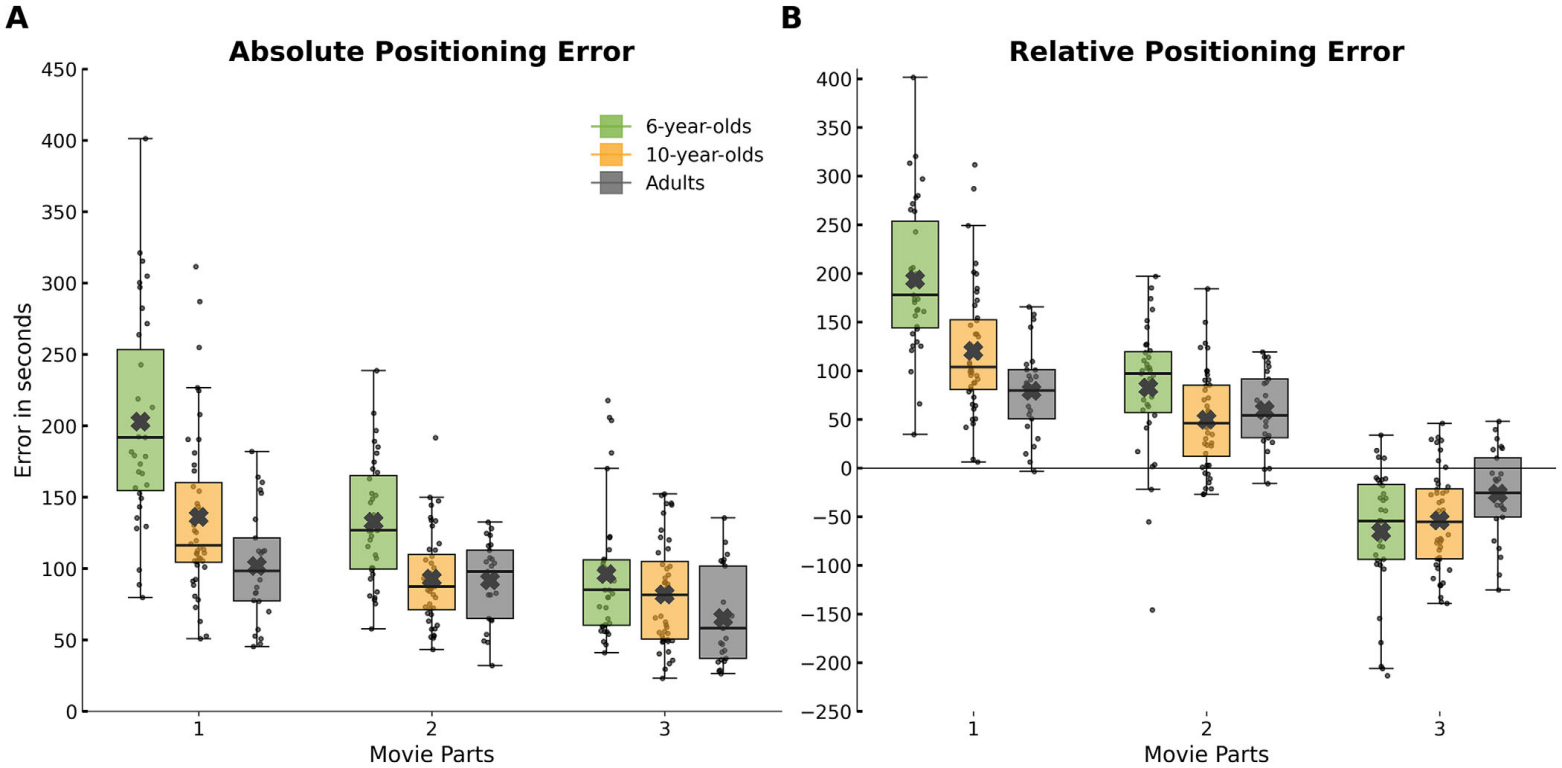
Interaction between Age group and movie part in the timeline task. Boxplots show positioning error by movie segment (divided into three parts: beginning, middle, end), separately for absolute (A) and relative (B) error. Group averages (black crosses) are displayed for each combination of age group and segment. Colors indicate age groups.

The experimental paradigm is illustrated in Figure 1. All participants completed the same set of tasks: (1) an encoding phase, where they watched a cartoon lasting approximately 11 min (Figure 1A); (2) a Timeline Task, where they placed clips from the cartoon on a timeline representing the duration of the episode, and rated each clip's importance for the story (Figure 1B); (3) a Number Line Task, where they placed numbers on a line ranging from 0 to 20 (Figure 1C); and (4) a Reordering Task, where they organized sets of cards into a sequential order so that they depict a coherent and meaningful figurative story (Figure 1D). The tasks were carried out in person using touch devices.

#### 2.2.1 Main Timeline Task

The Timeline Task comprised two phases: encoding and retrieval. During the encoding phase, participants viewed an episode of the animated series Robin Hood (Season 3, Episode 4, “The Furious Dragon”; duration: 10 min 57 s), with audio dubbed in Italian.

In the retrieval phase, participants first completed a familiarization session with the timeline interface used in the main task. The timeline appeared with the cursor initially centered, and participants were encouraged to touch and freely move the cursor to become acquainted with the interface. An experimenter was present throughout this phase to assist as needed, particularly for younger participants. During the familiarization, participants also performed two alignment trials to ensure understanding of the task mechanics. In each trial, two parallel horizontal timelines were presented: the cursor on the upper timeline was pre‐positioned (first at approximately 75%, then at 25%), while the lower cursor remained centered. Participants were instructed to touch and drag the lower cursor vertically to align it with the upper cursor. These trials ensured familiarity with spatial mapping before advancing to the main temporal memory task.

Following the familiarization phase, participants completed the main Timeline Task. They viewed a randomized sequence of short video clips (*N* = 66 trials; 60 experimental and 6 practice trials), each lasting 2 s, presented via the Qualtrics XM platform. Each trial began with the presentation of a clip at the center of the screen. Immediately afterward, a visual timeline appeared, representing the full duration of the previously viewed cartoon. A movable cursor was positioned at the center of the timeline, accompanied by the prompt: “Where does it go on the line?” Participants were instructed to touch and drag the cursor to the point on the timeline they believed corresponded to the moment in the episode from which the clip had been taken. Upon confirming their response, participants selected the “Next” button, located in the bottom right corner of the screen, to proceed. Subsequently, a new screen displayed a 7‐point Likert scale consisting of seven empty circles labeled from 1 to 7, alongside the prompt: “How important is it in the story?” Participants were asked to rate the narrative importance of the clip by touching the appropriate circle. After confirming their rating, a fixation cross appeared for 2 s, marking the inter‐trial interval before the next clip was presented. No time limits were imposed for either the temporal placement or importance rating tasks, allowing participants to respond at their own pace.

The clips were systematically extracted from the cartoon at fixed intervals of 7.3 s, starting from the beginning of the episode. The timeline interface was implemented as a continuous visual analog scale (VAS), divided into 720 segments, with each segment corresponding to 0.91 s of the episode's total duration. The VAS measured 14.5 cm in length on the touchscreen device.

#### 2.2.2 Number Line Task

The Number Line Task was implemented using a visual analog scale (VAS) ranging from 0 (left endpoint) to 20 (right endpoint), subdivided into 720 segments, maintaining the same resolution as the timeline task. Each integer unit on the scale corresponded to 36 segments. The task consisted of 20 trials, each corresponding to a target integer between 1 and 20.

On each trial, a target number (e.g., “8”) was displayed above the number line, accompanied by the prompt “Where does it go on the line?” presented below the scale. The cursor was initially positioned at the center of the line. Participants were instructed to touch and drag the cursor to the location they believed best represented the target number's position. Once satisfied with their placement, participants advanced to the next trial by selecting the “Next” button located at the bottom right corner of the screen. A fixation cross was then displayed for 2 s before the onset of the subsequent trial. No time constraints were imposed for responses. Although the task did not include a formal practice phase, the experimenter ensured that all participants fully understood the procedure before beginning the task.

#### 2.2.3 Reordering Task

The Reordering Task was adapted from the “Picture Arrangement” subtest of the WISC‐III (Wechsler 1991). Three stories were selected from the original 14 in the standardized subscale: Story 3 (four panels), involving a person crossing a river with a wooden plank; Story 5 (five panels), in which a boy falls into water from a dock; and Story 6 (four panels), depicting a cowboy entering a saloon. In each trial, the panels of a given story were presented simultaneously in a randomized order. Participants were instructed to reconstruct the logical sequence of events by writing numbers in boxes located beneath each panel. An experimenter was seated beside the participant and provided support if needed.

Before the main reordering task, participants completed a familiarization phase designed to introduce the picture sequencing procedure. This phase included three practice stories, each consisting of three panels representing simple everyday scenarios. These included: (1) a girl inserting a coin into a vending machine and drinking from the purchased can; (2) a child climbing stairs and sliding down a red slide; and (3) a dog stealing food from a picnic basket, which the couple later discovers to be empty. All practice items were adapted from the WISC‐III Picture Arrangement materials. For each practice trial, the three panels were presented in a scrambled order, resembling a wordless comic strip. A short instruction appeared above the images (e.g., “These pictures tell the story of a woman getting a drink. The images are out of order. Try to put them in the correct order so the story makes sense.”). Participants were instructed to assign a temporal order by writing the numbers 1, 2, and 3 in boxes located below the images, and then confirm their response using a designated button. After the initial attempt, the correct sequence was displayed along with brief explanatory captions beneath each image (e.g., “The woman takes coins from her purse”). Participants were asked to review the correct sequence carefully. The same set of scrambled images was then presented again, and participants were asked to reorder them a second time. This process was repeated for all three practice stories to ensure thorough comprehension of the task prior to test administration.

### 2.3 Data Analysis

#### 2.3.1 Timeline Task

Performance on the Timeline Task was quantified by calculating the deviation between the actual and expected clip positions on the timeline, expressed as both absolute error (unsigned distance) and relative error (signed distance). Absolute error reflects the overall magnitude of deviation between a participant's placement and the true clip position, regardless of direction; it indexes precision, providing a measure of how far off a response is from the correct location. In contrast, relative error reflects the direction of the deviation, indicating whether a systematic overestimation (clips positioned later than the correct position) or underestimation (clips positioned earlier than the correct position) is observed across subjects. Relative error has been used in previous studies to detect systematic biases in event placement and temporal memory (e.g., Frisoni et al. 2021, 2024), making it particularly useful for examining developmental differences in our sample.

To investigate potential modulations of performance as a function of narrative structure, a within‐subject factor Movie Part (Parts 1, 2, 3) was defined by dividing the 11‐min episode into three approximately equal temporal segments. This segmentation was purely temporal and pragmatic, rather than conceptual or scene‐based, and was not intended to reflect semantic or narrative boundaries. However, examining performance across these segments can provide insight into the role of narrative macrostructure: even younger children may possess a basic understanding of story structure (beginning, middle, end; Cutting and Cutting 2021), which likely aids the accurate placement of story endings—often prioritized in both event memory and narrative schemas (Alba and Hasher 1983; Bransford and Johnson 1972; Dooling and Christiaansen 1977; Schacter et al. 2007; Schank and Abelson 1977; Raykov et al. 2023). To examine the effects of age and narrative segment on temporal accuracy, two mixed‐model ANOVAs were conducted on absolute and relative error, with Age Group (6‐year‐olds, 10‐year‐olds, adults) as a between‐subjects factor and Movie Part (Parts 1, 2, 3) as a within‐subjects factor. Significant main effects and interactions were further explored using Tukey's HSD posthoc tests.

Temporal order memory was assessed by computing a Spearman rank‐order correlation between subjective and objective clip positions for each participant. The resulting individual correlation coefficients were Fisher *z*‐transformed to normalize the distribution. A one‐way ANOVA was then performed on these *z*‐values with Age Group as the between‐subjects factor.

To investigate the role of narrative importance on temporal precision, clips were categorized based on participants’ importance ratings into two levels: low importance (ratings 1–3) and high importance (ratings 5–7). Neutral ratings (score = 4) were excluded to maximize the contrast between conditions. Four participants (three 6‐year‐olds and one 10‐year‐old) were excluded from this analysis as they provided only high‐importance ratings. Within each age group, paired‐sample t‐tests compared absolute error for low‐ versus high‐importance clips. Additionally, to explore potential group differences in the perceived relevance of the clips, a one‐way ANOVA was conducted on average importance ratings with Age Group as the between‐subjects factor.

#### 2.3.2 Number Line and Reordering Task

For the Number Line Task, absolute and relative errors were computed using the same approach as in the Timeline Task. To determine whether developmental differences in timeline performance could be attributed to general difficulties in spatial magnitude estimation, a one‐way ANOVA was conducted on absolute error in number placement, with Age Group as the between‐subjects factor.

For the Reordering Task, performance was scored by assigning 1 point for each correctly ordered panel. Because the resulting data violated assumptions of normality, group comparisons were performed using the non‐parametric Kruskal–Wallis test.

#### 2.3.3 Multi‐Task Analyses

To assess whether developmental differences in temporal memory (Timeline Task) were influenced by visuospatial magnitude representation abilities (Number Line Task), a one‐way Analysis of Covariance (ANCOVA) was performed. Timeline task performance served as the dependent variable, Age Group as the between‐subjects factor, and number line performance was included as a covariate. To facilitate direct comparison between the Timeline and Number Line tasks, an error ratio was calculated for each trial. This ratio was defined as the absolute deviation from the correct location divided by the total number of segments on the line (720), yielding a normalized index of performance.

Finally, exploratory Spearman correlations were computed to examine relationships between performance across the three tasks within each age group. These analyses, based on the error ratio for the Timeline and Number Line tasks, aimed to investigate potential developmental trajectories across distinct cognitive domains.

## 3 Results

### 3.1 Timeline Task—Precision and Bias

The ANOVA on absolute error in the Timeline Task revealed a significant main effect of Age Group [*F*(2, 104) = 31.30, *p* < 0.0001, partial *η* ^2^ = 0.38] and Movie Part [*F*(2, 208) = 60.05, *p* < 0.0001, partial *η* ^2^ = 0.37], as well as a significant Age Group × Movie Part interaction [*F*(4, 208) = 6.54, *p* < 0.0001, partial *η* ^2^ = 0.11]. The main effect of Age Group was driven by significantly higher absolute errors in 6‐year‐olds compared to both 10‐year‐old children (*p* < 0.0005, *d* = 0.57) and adults (*p* < 0.0005, *d* = 0.72), and by significantly greater errors in 10‐year‐olds compared to adults (*p* < 0.05, *d* = 0.23; see Figure 2A). These findings indicate a linear developmental progression in the precision of temporal memory, with accuracy increasing from early to late childhood and almost reaching adult‐like levels. However, visual inspection of Figure 2A suggests that the performance gap between 10‐year‐old children and adults was notably smaller than that between the two groups of children.

This convergence between older children and adults was further supported by the analysis of relative error, reinforcing the view that primary school age represents a critical developmental window for temporal memory acquisition. The ANOVA on relative error revealed significant main effects of Age Group [*F*(2, 104) = 5.91, *p* < 0.005, partial *η* ^2^ = 0.11] and Movie Part [*F*(2, 208) = 364.20, *p* < 0.0001, partial *η* ^2^ = 0.78], along with a significant Age Group × Movie Part interaction [*F*(4, 208) = 21.22, *p* < 0.0001, partial *η* ^2^ = 0.29]. The main effect of Age Group was accounted for by a stronger tendency to overestimate clip positions in the 6‐year‐old group compared to both 10‐year‐old children (*p* < 0.01, *d* = 0.30) and adults (*p* < 0.05, *d* = 0.27). In contrast, no significant differences in the direction of error (i.e., overestimation vs. underestimation) were observed between the 10‐year‐old and adult groups (Figure 2B). Although Figure 2B suggests an overall positive bias, this effect was not uniform across the episode. As detailed in Figure 3B, overestimation was specific to early and middle segments, whereas late segments were systematically underestimated, consistent with a central tendency bias.

The Age Group × Movie Part interaction for absolute error revealed distinct developmental trajectories in temporal memory (Figure 3A). Adults’ performance remained stable across all parts of the movie (*p* = n.s.), indicating uniform accuracy throughout the episode. In contrast, 6‐year‐old children exhibited significantly greater errors in Part 1, with accuracy improving for Part 2 and further for Part 3. Specifically, absolute errors were significantly larger for Part 1 compared to both Part 2 and Part 3 (*p* < 0.0001), and for Part 2 compared to Part 3 (*p* < 0.05). 10‐year‐old children also showed elevated error for Part 1 relative to Parts 2 and 3 (*p* < 0.0001), but no difference between Part 2 and Part 3 (*p* = n.s.). This pattern suggests a developmental reduction in early‐event misplacement: younger children showed a pronounced tendency to misplace events from the beginning of the film, whereas older children exhibited a similar but less marked bias. Notably, no significant group differences were observed for Part 3 (*p* = n.s.), indicating that all age groups placed the final movie segments with comparable and high precision.

The interaction between Age Group and Movie Part yielded a clear developmental trend also for relative error (Figure 3B). Post hoc comparisons showed that 6‐year‐olds significantly overestimated the timing of clips from Part 1 compared to both older groups (*p* < 0.0001). No significant differences were found between 10‐year‐old children and adults for Part 1 (*p* = n.s.), nor were there age‐related differences for Parts 2 or 3 (all *p* = n.s.). Within‐group analyses further clarified this effect: 6‐year‐olds overestimated clips from Part 1 relative to Parts 2 and 3 (*p* < 0.0001), and underestimated clips from Part 3 relative to the earlier parts (*p* < 0.0001). 10‐year‐olds exhibited a similar within‐group bias—overestimating early and underestimating late segments—but with reduced magnitude (*p* < 0.0001). In contrast, adults showed no significant difference between Part 1 and Part 2 (*p* = n.s.) but consistently underestimated Part 3 relative to both earlier segments (*p* < 0.0001). Despite lower absolute error for Part 3 across all age groups, the relative error data revealed a consistent underestimation bias for these final scenes. This indicates that while participants placed later events with greater accuracy, they still tended to anticipate their occurrence, reflecting a central tendency bias—a compression of responses toward the center of the sequence under temporal uncertainty (Frisoni et al. 2024). This bias was most pronounced in 6‐year‐olds, remained observable in 10‐year‐olds, and persisted—albeit attenuated—in adults. Part of this central tendency bias might be explained by the fact that, on every trial, the clicker initially appeared in the middle of the timeline, representing an attractor for the temporal judgment. However, previous studies using non‐central starting positions have reported similar effects (Frisoni et al. 2024), suggesting an alternative explanation for the phenomenon. Importantly, the magnitude of the bias decreased systematically with age—strongest in 6‐year‐olds, reduced in 10‐year‐olds, and weakest in adults—suggesting that the effect primarily reflects cognitive constraints related to temporal uncertainty rather than the cursor's initial position.

**FIGURE 4:**
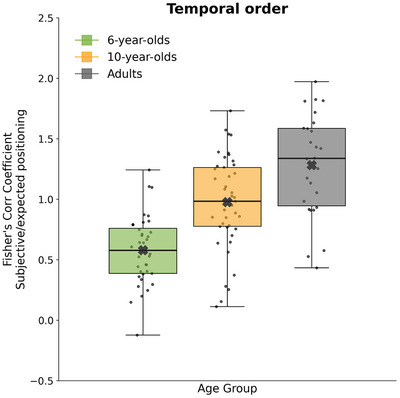
Temporal order as a function of age group in the timeline task. Boxplots show Fisher‐transformed Spearman correlation coefficients between subjective and objective clip positions, computed for each participant. Higher values indicate better preservation of temporal order. Colors indicate age groups.

The analysis of temporal order revealed a significant main effect of Age Group [*F*(2, 104) = 29.40, *p* < 0.0001, partial *η* ^2^ = 0.36]. Post‐hoc comparisons showed that younger children exhibited significantly lower correlation values than both older children (*p* < 0.0005, *d* = 1.17) and adults (*p* < 0.0005, *d* = 2.01), indicating a poorer ability to preserve the correct temporal order of events. Additionally, adults outperformed 10‐year‐olds (*p* < 0.005, *d* = 0.77), suggesting continued refinement of temporal sequencing abilities into adulthood (Figure 4).

### 3.2 Timeline Task—Effect of Clip Importance on Temporal Precision

**FIGURE 5:**
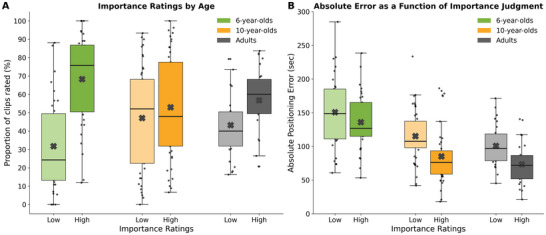
Relationship between importance and positioning accuracy per age group in the timeline task. (A) Boxplots showing the proportion of clips rated as high (Likert scores 5–7) or low (Likert scores 1–3) in importance across age group. Six‐year‐old children assigned overall higher importance ratings compared to older participants, reflecting reduced differentiation between more and less central narrative moments. Colors indicate age groups. (B) Absolute positioning error for clips rated as high versus low in importance. Ten‐year‐old children and adults were significantly more accurate in locating high‐importance compared to low‐importance clips, while 6‐year‐olds showed no reliable difference.

To examine how participants assigned narrative relevance to individual clips, we analyzed the proportion of clips rated as high (scores 5–7) or low (scores 1–3) in importance, excluding neutral ratings (score = 4). This proportional approach controlled for variability in the number of trials rated per participant, thus avoiding biases introduced by raw frequency counts (Figure 5A). Descriptive analyses revealed that 6‐year‐old children classified a significantly larger proportion of clips as high in importance (68.3% ± 24.4%) compared to 10‐year‐olds (52.9% ± 28.3%) and adults (56.8% ± 18.1%). Conversely, the youngest group assigned fewer clips to the low importance category (31.7% ± 24.4%) relative to the older groups.

To assess whether perceived importance influenced temporal precision, we compared absolute error for high‐ versus low‐importance clips within each age group (Figure 5B). Among 6‐year‐olds, temporal precision did not significantly differ between high‐importance clips (151 ± 53 s) and low‐importance (136 ± 42 s) clips [*t*(32) = 1.70, *p* = 0.098, Cohen's *d* = 0.30]. In 10‐year‐olds, absolute error was significantly lower for high‐importance clips (85 ± 41 s) than for low‐importance (115 ± 38 s) ones [t(43) = 4.62, *p* < 0.001, Cohen's *d* = 0.70]. A similar effect was observed in adults, who showed enhanced precision for high‐importance clips (73 ± 31 s) compared to low‐importance (101 ± 32 s) ones [*t*(25) = 4.32, *p* < 0.001, Cohen's *d* = 0.85]. Together, these findings indicate a developmental increase in the ability to leverage narrative importance as a cue for temporal memory. While older children and adults demonstrated significantly higher temporal precision for clips perceived as central to the story, younger children did not reliably benefit from this cue. This suggests that the ability to integrate event relevance into memory judgments is still emerging in early childhood. The age‐related strengthening of the association between importance and accuracy (Figure 5B) likely reflects a developmental shift in strategy use. Specifically, older participants may rely more effectively on an internal representation of the story's structure to guide memory placement, whereas younger children may lack a sufficiently mature narrative framework to meaningfully organize events in time.

### 3.3 Number Line and Reordering Task

**FIGURE 6:**
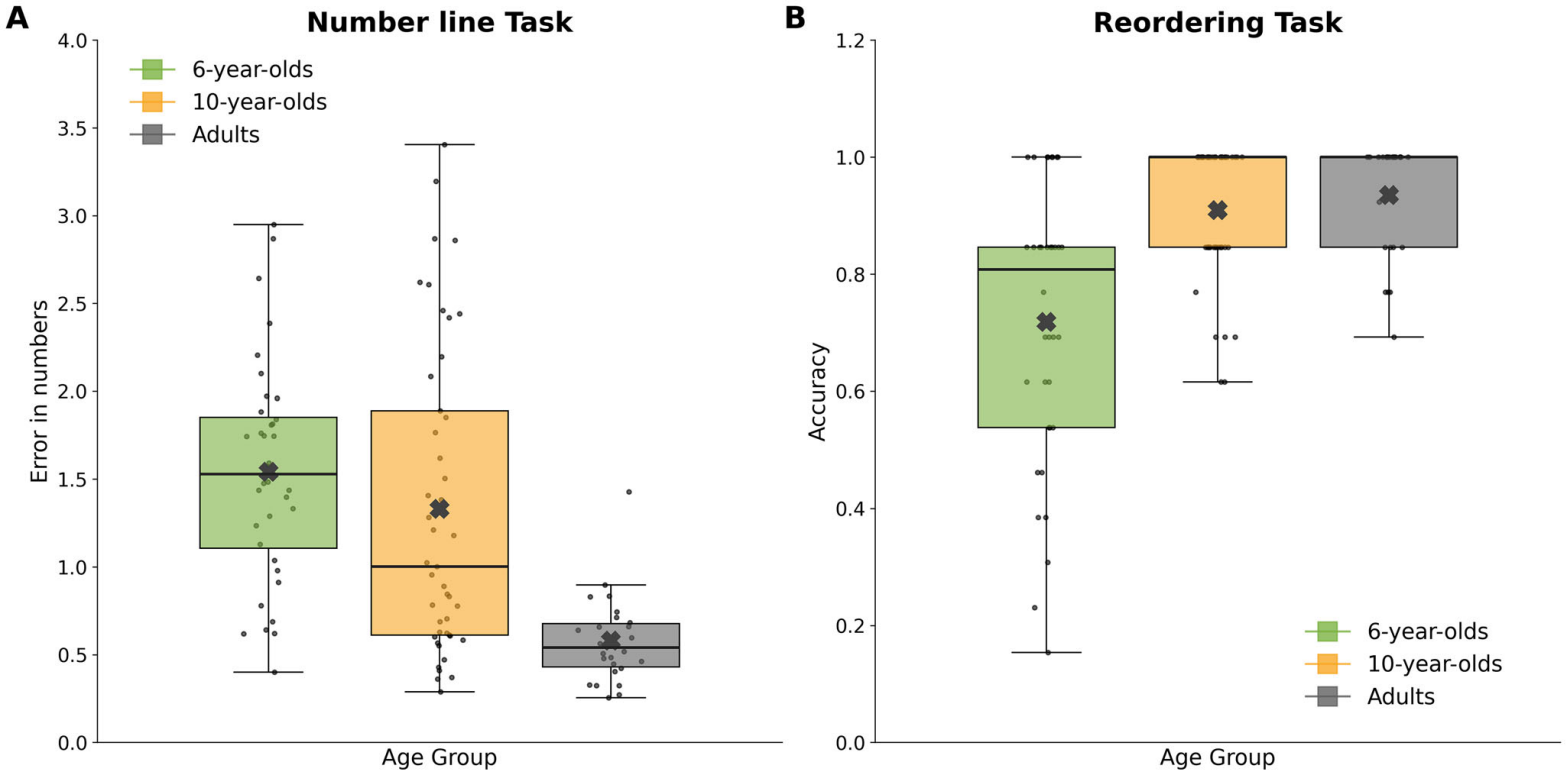
Figure 6 Number line and reordering task performance by age group. (A) Precision in the Number Line Task, shown as positioning error on the number line. (B) Accuracy in the Reordering Task, reflecting the correctness of the reconstructed sequence. Colors indicate age groups.

Part of the temporal error observed in younger children may reflect difficulties in using the timeline interface or in representing a mental spatial continuum more broadly. To evaluate this, we analyzed performance on the number line task, where absolute error served as the dependent variable in a one‐way ANOVA with Age Group as the between‐subject factor. The analysis revealed a significant main effect of Age [*F*(2, 104) = 15.79, *p* < 0.0001, partial *η* ^2^ = 0.23]. Post‐hoc tests indicated that adults were significantly more accurate than both 6‐year‐olds (*p* < 0.0005, *d* = 1.90) and 10‐year‐olds (*p* < 0.0005, *d* = 1.05), while no significant difference was observed between the two child groups (*p* = n.s.; Figure 6A). These results suggest that adults are generally more proficient in using number lines, whereas 10‐year‐olds have not yet developed a clear advantage over younger children in this domain. This pattern contrasts with the findings from the timeline task, in which 10‐year‐olds outperformed 6‐year‐olds. This dissociation suggests that, while older children may still struggle with the visuospatial demands of the timeline format, they no longer display the systematic temporal distortions characteristic of younger children, such as the pronounced overestimation bias seen in 6‐year‐olds (see Figure 2B).

Further support for this interpretation comes from the adapted WISC reordering task (Figure 6B), which assessed participants’ ability to arrange story elements in a logical temporal sequence. A Kruskal–Wallis H test revealed a significant difference in reordering performance across age groups [*H*(2, *N* = 107) = 22.07, *p* < 0.001], with a large effect size (*ε* ^2^ = 0.19). Post‐hoc comparisons showed that 6‐year‐olds scored significantly lower than both 10‐year‐olds (*p* < 0.001) and adults (*p* < 0.0005), indicating less developed narrative sequencing abilities. In contrast, no significant difference was found between 10‐year‐olds and adults (*p* = 1.000), suggesting that the ability to logically organize narrative content reaches near‐adult levels by late childhood. Taken together, these results indicate that younger children's temporal memory errors may partially reflect immature spatial and sequential processing abilities. However, the emerging dissociation between performance on spatial tasks and temporal memory suggests that older children begin to rely on different, possibly more abstract or semantic strategies to support memory placement—strategies that are not yet available to 6‐year‐olds.

### 3.4 Multi‐task Analyses

To determine whether developmental differences in timeline task performance could be attributed to difficulties with visuospatial representation, we conducted a one‐way ANCOVA with Age Group as the between‐subject factor and timeline accuracy (indexed by a normalized error ratio) as the dependent variable. Performance on the number line task was included as a covariate to control for spatial abilities. The use of a normalized error ratio allowed for direct comparability across temporal and spatial tasks. The assumptions for ANCOVA were met, as there was a significant positive correlation between timeline and number line performance across participants (Spearman's *ρ* = 0.45, *p* < 0.0001), confirming a linear relationship between the covariate and dependent variable. Additionally, the interaction between Age Group and the covariate was non‐significant (*p* = 0.585), satisfying the assumption of homogeneity of regression slopes. The ANCOVA revealed a significant main effect of Age [*F*(2, 103) = 20.86, *p* < 0.001, partial *η* ^2^ = 0.29], as well as a significant effect of number line performance [*F*(1, 103) = 8.56, *p* = 0.004, partial *η* ^2^ = 0.08]. Bonferroni‐corrected post‐hoc comparisons showed that 10‐year‐old children performed significantly better than 6‐year‐olds (*p* < 0.001), and that adults outperformed 6‐year‐olds (*p* < 0.001), but did not differ significantly from 10‐year‐olds (*p* = 0.79). These results indicate that developmental improvements in timeline accuracy are not fully explained by spatial abilities, particularly during the primary school years.

Additional Linear Mixed Model (LMM) analyses, which included Number Line and Reordering task performance as covariates, revealed a comparable developmental pattern (see Supplementary Analysis S1 in the Supplementary Materials). The model included Age Group and Gender as fixed effects and revealed a significant main effect of Gender: males showed higher temporal precision than females. However, there was no interaction between Gender and Age Group, indicating similar developmental trajectories for male and female participants.

**FIGURE 7:**
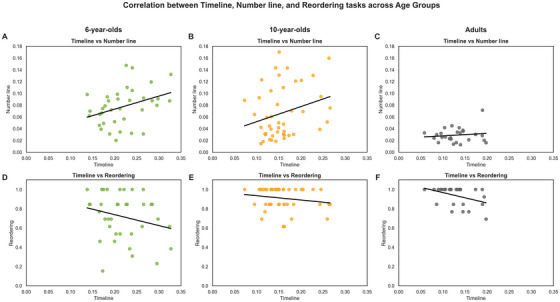
Correlations between timeline, number line, and reordering task across the three age groups. Panels (A)–(C) show the relationship between individual errors in the Timeline and Number Line tasks for 6‐year‐olds (A), 10‐year‐olds (B), and Adults (C). Panels D–F display the relationship between errors in the Timeline task and accuracy in the Reordering task for the same age groups: 6‐year‐olds (D), 10‐year‐olds (E), and Adults (F). Each point represents a participant. Colors indicate age groups, and black regression lines highlight the correlation trends.

To further explore cross‐task relationships, we conducted exploratory Spearman correlations between performance on the timeline, number line, and reordering tasks within each age group. Among 6‐year‐olds, we found a significant correlation between error ratios on the timeline and number line tasks (*r* = 0.39, *p* < 0.05; Figure 7A), suggesting that early temporal memory is closely linked to spatial‐numerical representations. This association weakened and became non‐significant in 10‐year‐olds (*r* = 0.27, *p* = 0.08; Figure 7B) and disappeared entirely in adults (*r* = –0.01, *p* = 0.96; Figure 7C). In contrast, timeline and reordering task performance were significantly correlated only in adults (*r* = –0.45, *p* < 0.05; Figure 7F), but not in 6‐year‐olds (*r* = –0.20, *p* = 0.22; Figure 7D) or 10‐year‐olds (*r* = –0.18, *p* = 0.24; Figure 7E).

Complementary analyses using LMM (see Supplementary materials), which included Number Line and Reordering performance as covariates, confirmed that developmental differences in timeline accuracy persisted even when controlling for spatial and narrative abilities. This suggests that age‐related improvements in temporal memory cannot be fully accounted for by maturation of spatial or sequencing skills alone (cf. the performance at the Number Line and Reordering tasks). The developmental shift observed here indicates that temporal cognition becomes increasingly dissociated from spatial‐numerical processing and more closely aligned with higher‐order narrative and inferential abilities in later stages of development. It is important to note, however, that nearly 50% of participants reached the maximum score (i.e., a perfect score of 1) on the reordering task, indicating a ceiling effect. This may have limited the sensitivity of the correlation analysis, particularly in older age groups, and should be considered when interpreting these findings.

## 4 Discussion

The present study investigated the development of temporal memory for complex events across childhood by comparing performance to that of adult participants. Children aged 6 and 10 years, along with young adults, viewed an 11‐min cartoon episode and were subsequently asked to place video clips extracted from the cartoon along a timeline corresponding to the episode's duration. To assess related cognitive abilities, participants also completed a number line task and a shortened version of the Picture Arrangement subscale from the Wechsler Intelligence Scale for Children (WISC‐III). The results highlight primary school age as a crucial period for the development of temporal memory. Temporal precision improved significantly between the ages of six and ten, with 10‐year‐olds' performance almost approaching that of adults, particularly in the absence of systematic directional biases. In contrast, 6‐year‐olds consistently overestimated the placement of early events, indicating a robust temporal bias. A central tendency bias—reflecting increased temporal uncertainty—was observed across all age groups but was most pronounced in younger children. Importantly, although 6‐ and 10‐year‐olds performed similarly on the number line task, only the older group showed enhanced accuracy in the timeline task, suggesting that temporal memory development involves more than visuospatial skills. This interpretation was supported by ANCOVA analyses, which confirmed that age‐related improvements in timeline accuracy remained significant even when controlling for performance in the number line task. In addition, both older children and adults placed high‐importance events more accurately, indicating that the ability to leverage narrative structure as a mnemonic scaffold emerges during the school years. Finally, correlational analyses revealed a developmental shift in the cognitive foundations of temporal memory: whereas younger children's performance was linked to spatial‐numeric abilities, adult performance was more closely associated with narrative reasoning, as measured by the Reordering Task. Together, these findings point to a developmental transition from early, spatially grounded temporal representations to more abstract, story‐based frameworks, marking a significant milestone in the maturation of episodic memory and event understanding.

### 4.1 The Development of Temporal Memory During Primary School

Our results indicate that children show measurable developments in temporal memory during the primary school period, consistent with prior findings showing that children's capacity for temporal reconstruction improves substantially across childhood (Friedman 1991; Friedman and Lyon 2005; Pathman, Larkina et al. 2013). We observed a robust age effect on temporal order memory, with younger children performing significantly worse than both older children and adults, and adults outperforming older children. These findings reveal a clear developmental trajectory in which the ability to situate complex events in time continues to improve from early childhood through adolescence and into early adulthood. This pattern aligns with research by Bettencourt et al. (2021), who documented age‐related improvements in memory for temporal context between both seven‐ to nine‐ and ten‐ to twelve‐year‐olds, and between children and young adults. Such developmental gains have been observed in both autobiographical and experimental contexts (Friedman and Lyon 2005; Pathman, Larkina et al. 2013; Jack et al. 2016), suggesting that the maturation of temporal memory is broad and generalizable, rather than limited to specific task types or content domains. This latter aspect is probably linked to even more general maturation of metacognitive and metamemory skills within these core ages (e.g., Forsberg et al. 2021; Pedale et al. 2022).

Memory for time, including both temporal order and temporal context, follows a protracted developmental course. While sensitivity to event sequences emerges in infancy (Bauer et al. 2007), this ability undergoes continuous refinement throughout middle childhood and adolescence (Canada et al. 2020; Pathman, Doydum et al. 2013; Picard et al. 2012). Temporal context memory develops more gradually and remains comparatively understudied, despite its foundational role in episodic and autobiographical recall. From early to middle childhood, children become increasingly adept at situating events on both arbitrary (e.g., experimental timelines) and conventional (e.g., calendar‐based) time scales (Friedman 1991; Pathman and Ghetti 2014). For instance, Pathman and Ghetti (2014) found that the ability to identify when events occurred improved significantly between ages 7 and 10, with continued gains into young adulthood. This extended developmental trajectory likely reflects the maturation of neural systems that support temporal binding and sequencing, particularly the hippocampus and prefrontal cortex, as well as the growing ability to leverage temporal patterns during memory retrieval (Friedman 1993, 2004). Supporting this view, prior work has shown that although preschoolers struggle with temporal inference, first and third graders are already capable of using temporal regularities to infer the timing of past events (Friedman and Lyon 2005), emphasizing the importance of the early school years in this domain.

Our findings contribute to this literature by demonstrating age‐related improvements in both the localization of events in time and the retrieval of correct event sequences. This dual advancement reinforces the view that the development of temporal memory unfolds across multiple complementary dimensions. While refinement continues into adolescence, our results underscore that the primary school years represent a pivotal window during which the foundational abilities that support complex temporal cognition undergo significant and measurable growth.

### 4.2 Central Tendency Bias and Temporal Representation

The analysis of relative error further supports the conclusion that temporal memory in older children resembles that of adults. While 6‐year‐olds showed a marked tendency to overestimate the timing of events, a reduced directional bias (over‐ vs. underestimation) was observed in the two older groups (i.e., 10‐year‐olds and adults), which did not differ from each other. Although older children were less accurate than adults in absolute terms, the reduction in the overestimation bias suggests a shift toward more adult‐like temporal estimation strategies by the end of primary school. These findings align with prior research showing that reconstructive temporal abilities depend on the integration of episodic memory with growing semantic knowledge of time (e.g., routines, calendars) (Friedman 1978, 1986; Friedman and Laycock 1989; Friedman and Lyon 2005), a process that appears to undergo significant refinement during this developmental period.

Closer inspection revealed that younger children were especially prone to overestimating the placement of early movie events—a bias reduced but still present in older children. In contrast, no group differences emerged for later events, where all age groups performed relatively well. This suggests that even younger children may possess a basic understanding of narrative macrostructure (beginning, middle, end; Cutting and Cutting 2021), which likely aids the accurate placement of story endings—often prioritized in both event memory and narrative schemas (Alba and Hasher 1983; Bransford and Johnson 1972; Dooling and Christiaansen 1977; Schacter et al. 2007; Schank and Abelson 1977; Raykov et al. 2023). Although absolute error was lowest for final events across groups, relative error analyses revealed a consistent underestimation bias—participants tended to place these events slightly earlier than their actual occurrence. This likely reflects a central tendency bias, in which uncertainty compresses responses toward the center of the timeline (Frisoni et al. 2024). The bias was strongest in 6‐year‐olds, attenuated in 10‐year‐olds, and persisted—though weakly—in adults. This gradient suggests that central tendency is a general cognitive heuristic, not limited to childhood. This pattern resembles a central tendency (Jazayeri and Shadlen 2010; Tal‐Perry and Yuval‐Greenberg 2022), whereby temporally uncertain events are reconstructed as closer to the center of a sequence. Importantly, this effect reflects allocentric reconstruction of narrative time rather than egocentric judgments relative to the present moment. One possible explanation for this phenomenon is that younger children encoded the first events of the narrative less efficiently as they either lacked a narrative schema or lacked sufficient attentional engagement. Another explanation is that younger children are more sensitive to longer retention intervals, which grows proportionally as the story unfolds.

Together, these findings highlight the persistence of central tendency bias across development, while also demonstrating its developmental modulation. As children's narrative understanding and temporal reasoning mature, they appear to rely less on heuristic strategies and more on structured temporal representations.

### 4.3 Importance of the Event as a Temporal Cue

Narrative importance has been shown to enhance long‐term memory, making crucial events more easily retrievable and more precisely located in time (Friedman 1993, 2004). According to schema theory, elements that align with a familiar narrative structure are more likely to be remembered (Alba and Hasher 1983). Prior research demonstrates that memory for stories is influenced by the hierarchical importance of events, their causal‐temporal relations, and the individual's knowledge of narrative organization (e.g., Rumelhart 1975; Rumelhart and Ortony 1977; Kintsch and van Dijk 1978; Thorndyke 1977; Mandler and Johnson 1977). Notably, recall and summarization studies show that participants reliably retrieve propositions rated as highly important, regardless of their position in the sequence, indicating that importance serves as a cognitive anchor for memory retrieval (Johnson 1970; Kintsch 1974; Thorndyke 1977; Yekovich and Thorndyke 1981).

To test whether importance also enhances temporal precision, we correlated individual importance ratings with absolute placement error. Older children and adults placed high‐importance clips significantly more accurately, whereas younger children did not show a reliable benefit. It is also possible that the limited benefit of importance cues for 6‐year‐olds reflects a tendency to broadly rate many clips as highly important, rather than an inability to use the cue per se. This distinction suggests that developmental differences in temporal precision may partly arise from age‐related differences in evaluating narrative relevance. This developmental shift suggests that the ability to use narrative importance as a cue for temporal localization consolidates during the primary school years. Our findings are consistent with those of Poulsen et al. (1979), who found that even four‐ and 6‐year‐olds recall story elements more easily when they form a coherent, causally linked sequence. However, while younger children may possess basic story schemas, older children and adults appear to use these schemas more strategically to integrate, infer, and temporally organize events.

In summary, our results point to a progressive development in the use of narrative importance as a mnemonic scaffold. By around ten years of age, children demonstrate adult‐like narrative organization, as seen in their accurate story reordering, comparable performance on the Reordering Task, and clear links between importance and temporal precision. However, they still show developmental limitations in fine‐grained temporal accuracy and event sequencing, indicating that while narrative understanding becomes well established in late childhood, other components of temporal memory continue to develop into adolescence.

### 4.4 The Transition From Spatial to Narrative Representation of Time

Our findings are consistent with a developmental trajectory whereby early temporal memory relies more heavily on spatial representations, whereas older children and adults increasingly utilize narrative‐based strategies. However, evidence of a complete shift remains inconclusive, as spatial and narrative abilities only partially account for age‐related differences. Stronger causal or longitudinal evidence is required to confirm this hypothetical transition scheme. Behaviorally, adults outperformed older children in temporal ordering tasks, yet 10‐year‐olds began to show adult‐like patterns, indicating the emergence of more mature encoding/retrieval strategies (cf. Figure 4). Older children also demonstrated sensitivity to narrative salience and were able to reorganize story elements logically, suggesting a transition toward conceptually driven event structuring (Friedman 2003; Friedman and Lyon 2005). This developmental trajectory is further supported by correlation analyses. Among 6‐year‐olds, performance on the timeline and number line tasks was significantly correlated, indicating reliance on shared spatial representations. This relationship diminished in 10‐year‐old children and disappeared in adults. Conversely, only adults showed a significant link between timeline accuracy and reordering task performance, suggesting that temporal memory becomes increasingly linked to higher‐order cognitive functions, such as inferential reasoning and narrative comprehension (Bonato et al. 2016; Orbach and Lamb 2007).

Research supports the view that space–time mappings are shaped by cultural and experiential factors, including literacy direction and embodied interactions (Tversky et al. 1991; Ouellet et al. 2010). Neuropsychological evidence also highlights a spatial basis for time. For example, adults with left hemispatial neglect show deficits in reasoning about past events (Saj et al. 2014), pointing to a neural link between spatial and temporal processing. This has practical implications, particularly in forensic contexts, where children are often expected to provide temporally specific accounts of recurring events (Powell, Roberts, Guadagno, 2007; Guadagno and Powell, 2014). However, since the ability to do so reliably only emerges around ages 8 to 10 (Friedman and Lyon 2005), reports by younger children should be considered with caution. Tools like pictorial timelines have been used to support children's temporal judgments, with 7‐ to 8‐year‐olds showing the highest concordance with parental reports and four‐year‐olds the lowest (Friedman 2003). Interestingly, the developmental shift observed in temporal cognition mirrors changes in numerical reasoning. Between kindergarten and sixth grade, children transition from logarithmic to linear number mappings, reflecting growing familiarity with symbolic systems (Siegler and Booth 2004; Siegler and Opfer 2003). These parallel shifts suggest a domain‐general progression from perceptual, analog representations to structured, conceptually mediated frameworks across both number and time.

### 4.5 Study Limitations

This study faced some constraints due to limitations set by the participating schools, especially regarding the total testing time. We prioritized the timeline task to ensure its completion and avoid fatigue. As a result, the timeline task was always administered first, and tasks followed a fixed sequence. For the same time restrictions, control tasks had fewer trials than the timeline task. We note that performance on the final task (Reordering) was generally high, indicating that fatigue did not negatively impact later measures.

Another limitation pertains to the response modality. Although the visual analogue scale (VAS) is a sensitive measure of temporal memory performance, we identified a potential developmental confound related to the maturation of the mental line. Future studies should consider alternative, but equally sensitive, methods for assessing internal temporal representations that do not depend on visuospatial transformation skills or basic sensorimotor abilities.

A further issue is represented by stimulus generalizability. Our study employed a single cartoon episode, a common approach in behavioral and neuroimaging studies of narrative memory (Furman et al. 2007; Kwok et al. 2014; Chen et al. 2017). Future work should test whether the developmental pattern described in the present study generalizes across diverse narrative types, including non‐fiction content, shorter or longer durations, or different presentation formats. The variation of narrative type might be accompanied by an assessment of the role of event boundaries for temporal memory. Previous research has started to investigate the issue (Montchal et al. 2019; Frisoni et al. 2021), but we note that a boundary analysis ideally relies on spontaneous event segmentation provided by independent raters, which can be problematic in a developmental framework.

Future work should also examine the role of event boundaries in developmental temporal memory. A theory‐driven boundary analysis would ideally rely on spontaneous event segmentation provided by age‐appropriate independent raters (cf. Montchal et al. 2019; Frisoni et al. 2021), which was beyond the scope of the present study.

Finally, further research should aim to disentangle the semantic, absolute positioning of events within the film's timeline from egocentric components, such as relative order judgments or spatial‐temporal distance relative to the self. Clarifying this distinction would enhance the understanding of the cognitive mechanisms driving temporal memory development.

## 5 Conclusions

Our findings highlight primary school years as a key period for developing temporal memory of complex events, reflecting a shift from spatially grounded to more abstract, narrative‐based temporal cognition. While 10‐year‐olds demonstrate adult‐like sensitivity to narrative importance in event placement, they still commit errors in event timing and sequencing, indicating ongoing refinement beyond late childhood.

Future research should investigate factors that accelerate this developmental trajectory and explore individual differences in temporal memory acquisition during primary school. Extending the developmental window would also clarify distinctions in temporal reconstruction abilities between children and adolescents.

## Conflicts of Interest

The authors declare no competing financial interests.

## Supporting information




**Supporting File 1**: desc70160‐sup‐0001‐SupMat.docx

## Data Availability

Data that support the findings of this study are available on request from the corresponding author.
