# Supplementary material for: The Development of Temporal Memory for Complex Events
Source: Dev Sci. 2026 Mar 3;29(3):e70160. doi: 10.1111/desc.70160 (PMC12957903; doi:10.1111/desc.70160)
Supplement: Supplementary file 1 — Supporting File 1: desc70160‐sup‐0001‐SupMat.docx [file DESC-29-e70160-s001.docx]

## **Supplementary Materials**

### **Supplementary Analysis S1**

**Linear Mixed Model Controlling for Spatial and Narrative Abilities**

To assess the robustness of the developmental effects reported in the main analyses, we conducted an additional Linear Mixed Model (LMM) analysis including individual differences in spatial and narrative abilities as covariates.

The model was fitted at the trial level, with timeline task accuracy (normalized error ratio) as the dependent variable. Age Group (6-year-olds, 10-year-olds, adults) and Gender were included as fixed effects, together with their interaction. Performance on the Number Line task and on the Reordering task were included as z-standardized continuous covariates. Participants were included as random intercepts to account for repeated observations. Model inference was based on Type III tests with Satterthwaite’s approximation for degrees of freedom (Kuznetsova et al., 2017).

The LMM revealed a significant main effect of Age Group [*F*(2, 99.00) = 11.36, *p* < .001], indicating that the developmental differences in timeline performance observed in the primary analyses persist when controlling for spatial magnitude representation and narrative sequencing abilities. Significant main effects were also observed for Number Line performance [*F*(1, 99.00) = 6.48, *p* = .012], and for Reordering task performance [*F*(1, 99.00) = 6.42, *p* = .013], suggesting that individual variability in these abilities is associated with temporal precision across participants.

A modest main effect of Gender was also observed [F(1, 99.00) = 7.45, p = .008], with males showing slightly higher temporal precision than females. The Age × Gender interaction was not significant [F(2, 99.00) = 0.55, p = .577], indicating comparable developmental trajectories across genders. Sample sizes were unequal (N = 39 males, N = 68 females), and the effect should therefore be interpreted cautiously as exploratory.

This supplementary LMM addresses a complementary question to the primary ANCOVA-based analyses reported in the main text. Both Number Line and Reordering task performances are developmentally sensitive abilities and may partially mediate age-related differences in temporal memory. Consequently, including them as covariates provides a conservative estimate of age effects. The persistence of a significant Age Group effect under these conditions indicates that developmental differences in temporal memory cannot be fully explained by improvements in spatial or narrative abilities alone.

With respect to the Reordering task, although no group-level differences were observed between older children and adults in the main analyses, its significant contribution in the LMM suggests that individual variability in narrative sequencing ability predicts timeline accuracy when considered across the full sample. Given the reduced variability in older participants, this result should be interpreted with caution.

Overall, this supplementary analysis supports the robustness of the developmental pattern reported in the main text while clarifying the role of spatial and narrative abilities in explaining individual differences in temporal memory.

References

Kuznetsova A, Brockhoff PB, Christensen RH (2017) lmerTest package: tests in linear mixed effects models. J Stat Softw 82:1-26. https://doi.org/10.18637/jss.v082.i13
